# Plant stress responses compromise mutualisms with *Epichloë* endophytes

**DOI:** 10.1093/jxb/erac428

**Published:** 2022-10-30

**Authors:** Daniel A Bastías, Pedro E Gundel

**Affiliations:** AgResearch Limited, Grasslands Research Centre, Palmerston North 4442, New Zealand; IFEVA, CONICET, Universidad de Buenos Aires, Facultad de Agronomía, Buenos Aires, Argentina; Centro de Ecología Integrativa, Instituto de Ciencias Biológicas, Universidad de Talca, Talca, Chile; University of Warwick, UK

**Keywords:** Antioxidant, endophytes, mutualism, phytohormones, plant–endophyte symbiosis, ROS

**Plants commonly form mutualistic associations with fungal endophytes. We put forward the hypothesis, with supporting evidence, that certain plant physiological responses to stress [i.e. phytohormones and reactive oxygen species (ROS)] change the symbiosis between plants and *Epichloë* endophytes from mutualistic to parasitic. The change in symbiosis outcome would be explained by the negative effects of the plant physiological responses on the endophyte performance. Furthermore, we posit that endophytes may protect the mutualism by the induction of plant defence hormone responses and antioxidants.**

The outcomes of plant–microbe symbioses are considered mutualistic when both partners obtain benefits, parasitic when one partner receives benefits and the other is harmed, or commensalistic when one partner obtains benefits and the other is not affected (Newton *et al*., 2010). A remarkable example of mutualism is the association between cool-season grasses and asexual fungal endophytes of the genus *Epichloë* (Schardl *et al*., 2004). These endophytes increase plant fitness by producing bioactive secondary metabolites, most commonly alkaloids, that boost plant defence against herbivores and by increasing antioxidant contents that enhance plant tolerance to oxidative stress caused by the accumulation of ROS (Hamilton *et al.*, 2012; Bastias *et al.*, 2017). In turn, plants provide the fungus with a place to live, photosynthates (and nutrients), and a propagation mode (asexual endophytes are transmitted through seeds) (Schardl *et al*., 2004).

Evolutionary theory predicts that associations of plants with vertically transmitted microbes should be mutualistic since the survival of the symbionts is entirely dependent on the fitness of their plant hosts (Ewald, 1987). However, even in these mutualistic associations, the fitness of symbiotic plants, relative to that of non-symbiotic plants, can be transiently compromised under certain environmental circumstances (Newman *et al*., 2022). This fitness dependency in symbiotic plants on environmental conditions can change the symbiosis outcome from mutualistic to parasitic (Hoeksema *et al*., 2010; Chamberlain *et al*., 2014). In the case of plants associated with asexual *Epichloë* endophytes, these associations can indeed turn into parasitic ones when grown in soils with low nutrient contents (Cheplick *et al.*, 1989; Ahlholm *et al.*, 2002; Cheplick, 2007; Wäli *et al.*, 2008). There is consensus that the reduced fitness in symbiotic plants (relative to endophyte-free plants) grown in soils with nutrient scarcity is due to the metabolic costs associated with the maintenance of endophytes (Cheplick *et al.*, 1989; Cheplick and Faeth, 2009).

Here, we propose that the symbiosis outcome in plants associated with endophytes can be compromised, in addition to plant resources, by the action of phytohormones and ROS that are induced by environmental stressors. At medium to high stress levels, the activation of defence-related phytohormones and/or ROS may change the symbiosis from mutualistic to parasitic due to the reduction in fitness of endophyte-symbiotic plants (relative to non-symbiotic plants) caused by the disruption in the endophyte-derived benefits and growth of the symbionts within plant tissues (Fig. 1). We describe experimental results showing that plant–*Epichloë* mutualisms were compromised by environmental stressors. Plant-*Epichloë* associations were used as symbiosis model since the fungi mainly form mutualistic associations with plants, can be vertically transmitted though seed, confer well-understood benefits to plants, and influence plant physiological responses. Our hypothesis contributes to improving the understanding of the effects of environmental stressors on mutualisms of plants associated with endophytes.

**Fig. 1.**
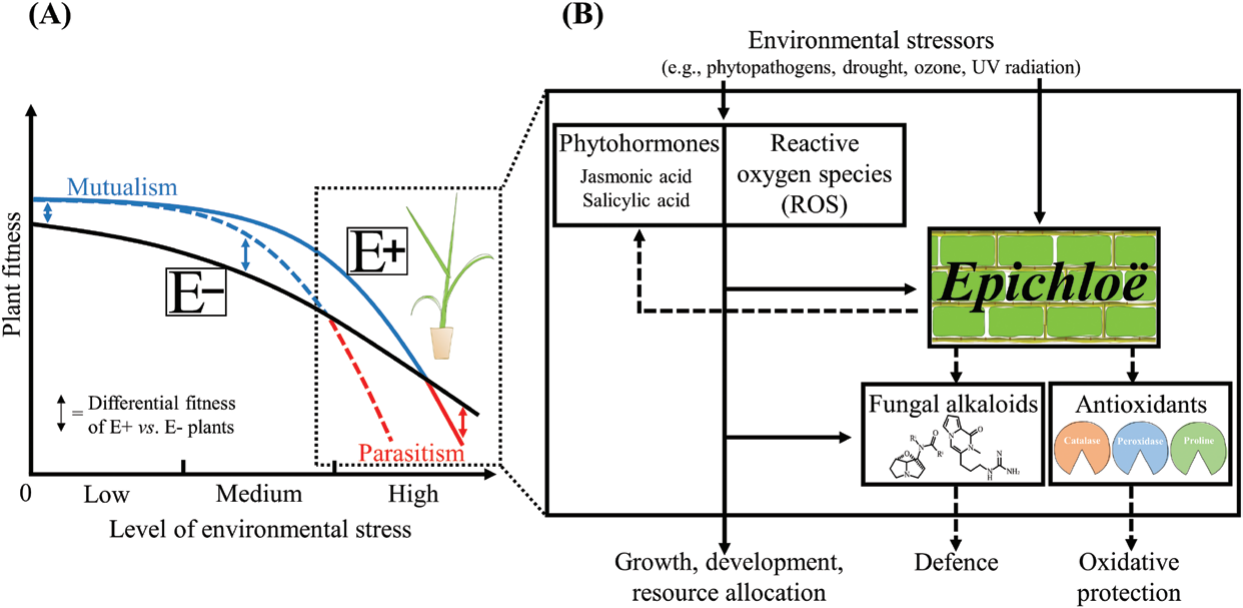
Proposed symbiosis outcome regulation by phytohormones and ROS in plants symbiotic with asexual *Epichloë* endophytes. (A) Model showing predicted fitness changes of endophyte-symbiotic (E+; blue/red lines) and non-symbiotic (E–; black line) plants across a range of environmental stress levels (any abiotic or biotic). The symbiosis outcome (i.e. whether the relationship is mutualistic, neutral, or parasitic) is determined by the comparison of predicted E+ and E– fitness lines at a given stress level (double-headed arrows). At low to medium stress, the symbiosis is mutualistic due to the higher fitness of E+ compared with E– plants (Schardl *et al.*, 2004; Bastías *et al.*, 2021). However, at medium to high stress, the symbiosis turns into parasitic due to the lower fitness of E+ relative to E– plants. The continuous and discontinuous lines for E+ plants refer to associations where the symbiont provides or does not provide mechanisms of direct protection against stresses, respectively. In cases where the endophyte provides stress protection, the change in symbiosis outcome would occur at higher stress levels than when the endophyte does not provide the protection. (B) The action of defence-related phytohormones and/or ROS can explain the parasitic relationships at high stress. Environmental stressors that activate defence-related hormone and ROS responses in plants affect the performance of *Epichloë* by disrupting the endophyte alkaloid production and/or fungal growth within plant tissues (Pozo *et al.*, 2015; Bastias *et al.*, 2017, 2022; Kayano *et al.*, 2018). Hormone- and ROS-mediated negative effects on endophyte performance may decrease the E+ fitness to a point that the symbiosis turns into parasitic. The direct and negative effects of stresses on endophytes also contribute to the change in symbiosis outcome (Gundel *et al.*, 2012). At the same time, endophytes can protect mutualisms in stress situations by stimulating defence-related phytohormone responses and enhancing antioxidant contents in plants (Hamilton and Bauerle, 2012; Kou *et al.*, 2021). In (B), solid and dashed lines indicate stress/plant and endophyte regulatory mechanisms, respectively.

## Phytohormones and ROS modulate the outcome of plant–endophyte symbioses

Defence-related phytohormones can reduce the *Epichloë*-based production of alkaloids, diminish the herbivore resistance of symbiotic plants, and compromise the mutualism (Fig. 1). The exogenous application of salicylic acid (SA) or methyl jasmonate [MeJA; an activator of the jasmonic acid (JA) signalling pathway] to endophyte-symbiotic *Lolium multiflorum* plants reduced the concentrations of *Epichloë*-derived alkaloids within foliar tissues (Bastías *et al*., 2018a, b). A similar outcome in alkaloid concentrations was documented in endophyte-symbiotic *Festuca arundinacea* plants treated with MeJA (Simons *et al*., 2008). The reductions in alkaloid concentrations were associated with fitness depressions in host plants since endophyte-symbiotic plants exposed to the hormones exhibited lower levels of resistance to insects than symbiotic plants not treated with hormones (Simons *et al*., 2008; Bastías *et al*., 2018a, b). Furthermore, the endophyte-mediated change in the balance of defence-related hormones could also influence the plant resistance against phytopathogens, as reduced SA levels in *Epichloë* symbiotic plants have been generally associated with susceptibility toward biotrophic pathogens (Bastias *et al.*, 2017). Interestingly, defence-related phytohormones can turn plant–*Epichloë* associations into parasitic interactions. *Spodoptera frugiperda* larvae showed higher biomass when fed on endophyte-symbiotic *L. multiflorum* plants treated with MeJA than on non-symbiotic plants either treated or not with the hormone (Bastías *et al*., 2018b). Since endophytes negatively affected the plant resistance to herbivores under the hormone treatment, this result suggests that the fungus exhibited, at least temporarily, a parasitic behaviour.

Environmental stressors that activate defence-related phytohormone responses can reduce the herbivore resistance of symbiotic plants and compromise the mutualism (Fig. 1). A short-term exposure of *L. multiflorum* plants to ozone, a gas known to induce SA-related responses (Sandermann *et al*., 1998), significantly reduced the resistance to aphids conferred by *Epichloë* endophytes (Ueno *et al.*, 2016). The effects of environmental stressors can, via the activation of plant defence-related hormones, change the outcome of plant–endophyte symbioses. Endophyte-symbiotic *Festuca pratensis* plants exposed to UV radiation, which induces JA-related defence responses (Qi *et al*., 2018), were more susceptible to *Schistocerca gregaria* locusts than their non-symbiotic counterparts, whereas the opposite was observed in symbiotic plants under ambient radiation (McLeod *et al*., 2001). Since the fungus negatively affected the plant resistance to herbivores under the UV treatment, this finding indicates that the endophytes behaved parasitically within their plant hosts.

Defence-related phytohormones can also inhibit the *Epichloë*-mediated stimulation of plant growth, reduce the fitness of symbiotic plants, and compromise the mutualism. Biomass gain in *Achnatherum sibiricum* plants due to the endophyte presence was eased when symbiotic plants were sprayed with MeJA. In this experiment, symbiotic plants treated with MeJA tended to show a lower foliar biomass than their non-symbiotic counterparts also treated with the hormone (overall, MeJA-exposed plants showed lower biomass gain than plants not exposed to the hormone) (Qin *et al*., 2019). Since the endophytes seemed to negatively affect the plant growth under the hormone treatment, we consider that in this experiment the fungus tended to show a parasitic behaviour.

ROS can disrupt the growth of *Epichloë* endophytes within plant tissues and compromise the mutualism (Fig. 1). Endophytes with a mutation in the fungal *NoxA* gene, which encodes a NADPH oxidase that produces ROS, exhibited an uncontrolled growth inside host plants that was associated with severely stunted (and sometimes lethal) phenotypes in symbiotic plants (Tanaka *et al*., 2006). Similar phenotypes in the fungus and their plant hosts were found in associations involving *Epichloë* with mutations in genes encoding proteins that regulate the function of the fungal NoxA NADPH oxidase enzyme (Kayano *et al*., 2018). These findings demonstrate that the fungal production of ROS is a key element in the maintenance of the mutualism between plants and endophytes.

Environmental stressors that increase ROS levels in plants can reduce the fitness of symbiotic plants and compromise the mutualism (Fig. 1). Besides affecting the SA signalling pathway, the tropospheric pollutant ozone can increase ROS-associated oxidative damage in *Epichloë*-symbiotic plants (Ueno *et al*., 2021a). The effects of environmental stressors, via an increase in ROS, can change the outcome of plant–endophyte symbioses. Endophyte-symbiotic *L. multiflorum* plants exposed to high ozone levels exhibited lower reproductive effort (the proportion between reproductive and shoot biomass) than non-symbiotic plants exposed to the same treatment (whereas the endophyte did not affect the reproductive effort of plants not exposed to ozone) (Ueno *et al*., 2021a). Since ozone reduced the fitness of endophyte-symbiotic plants to a greater extent than that of plants free of these endophytes, the ozone changed the endophyte behaviour from mutualistic to parasitic.

Environmental stressors can also directly affect the fungus, compromising plant–*Epichloë* mutualisms (Fig. 1). The herbicide diclofop-methyl, an acetyl-CoA carboxylase (ACCase) inhibitor, reduced the endophyte-derived insect herbivore protection (Gundel *et al.*, 2012). Considering that these plants were herbicide resistant and that the fungus may harbour ACCases (Hunkeler *et al.*, 2016), the direct herbicide effect on the endophyte metabolism could explain the reduction in the plant herbivore resistance.

## Endophytes endow plant hosts with mechanisms to protect the mutualism

*Epichloë* can prevent the change in the symbiosis outcome in situations of high stress levels by inducing defence-related hormone and antioxidant plant responses that increase plant fitness (e.g. Hamilton and Bauerle, 2012; Kou *et al*., 2021).

*Epichloë* endophytes can induce plant responses associated with defence hormones (i.e. SA and JA) that increase the fitness of symbiotic plants in situations of stress by phytopathogens (Fig. 1). The resistance level of *Achnatherum inebrians* against the phytopathogen *Blumeria graminis* was increased by the presence of endophytes that boosted plant defences by inducing SA-related responses (Kou *et al*., 2021). Similarly, enhanced resistance against *Curvularia lunata* pathogen was documented in *A. sibiricum* plants associated with endophytes that induced plant JA-related defence responses (Shi *et al*., 2020). In a distinct symbiotic system, enhanced resistance against the pathogen *Alternaria solani* was shown in *Solanum lycopersicum* plants symbiotic with *Serendipita indica* root endophytes that primed JA/ethylene host defence responses (Panda *et al.*, 2019).

*Epichloë* can also enhance antioxidant levels that generally increase the fitness of symbiotic plants under stressors that increase ROS-associated oxidative damage (Fig. 1) (Ueno *et al*., 2021b). Under water deficit, the biomass of *Elymus dahuricus* plants was increased by the presence of endophytes that enhanced contents/activities of several antioxidants that reduced the oxidative damage within plant tissues (Zhang and Nan, 2007). Similarly in the presence of phytopathogens, the biomass of *L. perenne* plants was also increased by endophytes that enhanced the contents/activities of several antioxidants within symbiotic plants (Ma *et al*., 2015).

## Conclusions and future perspectives

Here we have highlighted that the effects of environmental stressors can compromise the mutualism between plants and endophytes by the induction of plant physiological responses. Whereas phytohormone- and ROS-related responses were considered independent here, in reality the whole-plant response to stresses involves the interaction between both pathways (Overmyer *et al*., 2018). Further experiments may determine the effects of the interconnection between these signalling pathways in the regulation of plant–endophyte mutualisms. Furthermore, additional experiments may determine the relative importance of the endophyte-derived mechanisms of mutualism protection that were described in the present study (and possibly identify novel mechanisms).
